# Platelet-rich plasma-derived microRNA let-7a-5p alleviates knee osteoarthritis by regulating macrophage polarization and improving inflammatory microenvironment

**DOI:** 10.3389/fimmu.2026.1756467

**Published:** 2026-02-16

**Authors:** Qishan Li, Mengjie Wang, Dong Wang, Yaochi Nie, Xueyuan Sun, Lin Na, Dongmei Yan, Yuhang Ma, Hui Wang

**Affiliations:** 1 Department of Blood Transfusion, the Second Affiliated Hospital of Harbin Medical University, Harbin, China; 2 Scientific Research Centre, the Second Affiliated Hospital of Harbin Medical University, Harbin, China

**Keywords:** inflammatory microenvironment, knee osteoarthritis, let-7a-5p, macrophage polarization, MAPK8, platelet-rich plasma

## Abstract

**Background:**

Knee osteoarthritis (KOA) is closely associated with an imbalance in macrophage M1/M2 polarization within its inflammatory microenvironment. Platelet-rich plasma (PRP) has demonstrated therapeutic efficacy in KOA. Moreover, microRNAs (miRNAs) also play a protective or destructive role in the pathogenesis of KOA. This study aims to elucidate the molecular mechanisms by which PRP-related miRNAs ameliorate the inflammatory microenvironment to alleviate KOA.

**Methods:**

An *in vivo* KOA rat model was established via intra-articular injection with monosodium iodoacetate (MIA). Safranin O-fast green staining and hematoxylin and eosin (HE) staining were used to assess cartilage degeneration and synovial inflammation, respectively. Macrophage phenotype was analyzed by immunohistochemistry (IHC) and immunofluorescence (IF). Reverse transcription-quantitative polymerase chain reaction (RT-qPCR) was used to examine the expression of inflammatory cytokines. Chondrocyte anabolic and catabolic status was evaluated using IF and western blotting (WB). Bioinformatics analysis was employed to screen for differentially expressed miRNAs in PRP and Dual-luciferase reporter assay was conducted to verify that mRNA is a direct target for miRNA. Furthermore, we explored the biological functions of miRNA and mRNA by transfecting mimics and siRNA.

**Results:**

*In vitro*, PRP inhibited M1-type macrophage polarization while promoting M2-type polarization, leading to suppressed pro-inflammatory cytokine release and enhanced anti-inflammatory cytokine release, collectively reducing cartilage degeneration. We identified microRNA let-7a-5p using bioinformatic approaches and subsequently investigated its molecular mechanisms. Similar to PRP, let-7a-5p was found to regulate macrophage polarization, the release of inflammatory cytokine, and cartilage degeneration. Furthermore, we identified and experimentally validated MAPK8 as a target gene of let-7a-5p.

**Conclusion:**

PRP reshapes macrophage polarization by regulating the let-7a-5p/MAPK8 axis, thereby improving the inflammatory microenvironment of KOA and providing a potential new therapeutic target for KOA management.

## Introduction

Knee osteoarthritis (KOA), over half of all global osteoarthritis cases (1), is a chronic degenerative joint disease characterized by articular cartilage degeneration, synovial inflammation, and subchondral bone remodeling. Primary symptoms include pain and restricted mobility, which can lead to functional impairment in severe cases. Its prevalence is rising in tandem with societal aging and increasing obesity rates, significantly impairing patients’ quality of life and imposing a substantial socioeconomic burden (2).

In the past, KOA was considered a “wear and tear” disease mainly involving chondrocytes. However, increasing evidence suggests that various factors (synovial inflammation, macrophage polarization and subchondral bone remodeling) are involved in the pathogenesis of KOA (3, 4). Among these factors, synovial inflammation is one of the key risk factors that persist throughout the disease process. The synovium is primarily composed of macrophages and fibroblast-like synoviocytes (FLSs) (5). Joint inflammation triggers macrophage activation and polarization towards M1 phenotype, leading to the release of pro-inflammatory mediators (IL-1β, IL-6, and TNF-α), which establish a pro-inflammatory microenvironment and drive inflammatory responses (6). Furthermore, this pro-inflammatory state disrupts the balance between extracellular matrix (ECM) synthesis and chondrocyte degradation, ultimately leading to cartilage degeneration and loss of cartilage integrity. Damaged chondrocytes can trigger additional release of matrix metalloproteinases (MMPs) and inflammatory cytokine (7, 8), exacerbating synovitis and perpetuating the vicious cycle, further exacerbating KOA in the pro-inflammatory microenvironment.

Non-surgical treatment methods for KOA patients include physical therapy, intra-articular injection, and nonsteroidal anti-inflammatory drugs (NSAIDs), which can only alleviate pain symptoms but do not improve the degenerative changes of articular cartilage. In recent years, Platelet-rich plasma (PRP) has emerged as a promising treatment method in regenerative medicine.

PRP, an autologous blood product rich in growth factors, chemokines, and cytokines, plays a significant role in treating osteoarthritis and promoting wound healing (9, 10). Intra-articular injection of PRP has become an established therapeutic strategy for KOA. Not only can it alleviate pain symptoms, but more importantly, it can significantly mitigate inflammation, enhance angiogenesis, promote chondrocyte proliferation and differentiation, and exert protective effects on articular cartilage (11). Due to its favorable safety profile and minimal adverse reactions, PRP has gained widespread clinical application in recent years. Despite its established therapeutic efficacy, the precise molecular mechanisms underlying its actions remain incompletely elucidated.

MicroRNAs (miRNAs) are a class of small non-coding RNAs that have emerged as key post-transcriptional regulators of gene expression by binding to the 3′-untranslated regions (3′-UTRs) of target messenger RNAs (mRNAs). This regulatory capacity allows them to govern a wide array of biological processes, including those critical to the progression of osteoarthritis (OA) (12). For instance, a number of studies have demonstrated that miRNAs can exert either pro- or anti-inflammatory effects by modulating the expression levels of catabolic and anabolic genes (13, 14). A growing body of evidence has highlighted the crucial role of miRNAs in OA pathogenesis (15–18). For example, some studies have shown that specific nanomedicines can alleviate OA progression by suppressing M1-type macrophage polarization and promoting M2-type polarization via miRNA-mediated mechanisms (19). As a promising therapeutic agent for OA, PRP is known to contain a rich repertoire of miRNAs. However, the specific miRNAs within PRP that mediate its therapeutic effects, particularly in the context of modulating the inflammatory microenvironment, remain largely unexplored.

This study aims to identify and elucidate the role of a key PRP related miRNA in improving KOA and explore its therapeutic potential and molecular mechanisms in alleviating KOA.

## Materials and methods

### Animals and experimental design

Sprague-Dawley (SD) male rats (n=18, 6–8 weeks, 180–220 g) were provided by the Animal Experiment Center of the Second Affiliated Hospital of Harbin Medical University and were housed under pathogen-free conditions. All animal studies were performed in accordance with the ethical principles of animal protection and welfare, and approved by the Ethics Committee of the Second Affiliated Hospital of Harbin Medical University (Ethical review approval number: SYDW2024-024). The rats were randomly divided into three groups: control, KOA, and PRP groups. For the experimental design, SD rats were administered a single right knee joint intra-articular injection of monosodium iodoacetate (MIA) (injection dose: 2mg/50μl) (YuanYe S30680) at week 0 to establish KOA rat models. Two weeks after KOA rat models induction, rats were intra-articularly injected into the right knee joint with either saline or PRP, 50 μl for each injection, respectively, at weeks 2, 3, 4, and 5. All rats were sacrificed at week 6, and cervical dislocation was performed to ensure euthanasia. Knee joint samples were collected for subsequent histological analyses.

### PRP extraction

PRP was extracted from male SD rats (6–8 weeks, 180–220 g). The rats were anesthetized via intraperitoneal injection of 1% pentobarbital sodium solution (injection dose: 40mg/kg) (Sigam P3761). The abdominal skin was cut, the abdominal aorta was separated and exposed. Whole blood samples were collected and placed with the anticoagulant tubes containing sodium citrate, with a ratio of 9:1 (v/v). After sufficient mixing, the whole blood was centrifuged at 2400 rpm for 10 min. The resulting supernatant was then centrifuged for centrifugation at 3600 rpm for 15 min. PRP was obtained after discarding three-quarters of the supernatant. The platelet count in whole blood was 88.5 ± 9.1×10^4^ platelets/μl; in PRP, the count was approximately 3.8-fold higher at 333.7 ± 43.3×10^4^ platelets/μl. This concentration is consistent with the established standards for PRP preparation. Mixing PRP with 10% calcium gluconate in a ratio of 9:1 (v/v) to activate PRP. The mixture was incubated overnight at 4°C and subsequently centrifuged at 12,000×g for 20 min at 4°C. The supernatant was stored at a temperature of -80°C for later use.

### Construction of KOA rat models and Osteoarthritis scoring

The rats were anesthetized with 1% pentobarbital sodium solution (injection dose: 40mg/kg) (Sigam P3761), and the right knee of the hind leg was shaved and sterilized. MIA (injection dose: 2mg/50μl) (YuanYe S30680) was injected into the cavity of the right knee joint through the infrapatellar ligament. Histological sections of rat knee joints were stained with Safranin O-fast green to assess cartilage degeneration and with hematoxylin and eosin (HE) to evaluate synovial inflammation. Two independent, blinded observers performed the scoring to ensure objectivity. Cartilage degeneration was scored using the Osteoarthritis Research Society International (OARSI) grading system, which ranges from 0 (no change) to 7 (complete loss of uncalcified and calcified cartilage). Synovitis was evaluated using a four-point scoring system, where grades ranged from 0 (no synovitis) to 3 (marked synovial hypertrophy). The final scores were determined by averaging the results from the two observers.

### Chondrocyte isolation and culture

Articular cartilage was aseptically harvested from the knee joints of SD rats. The cartilage tissue was minced into small fragments (approximately 1 mm³). These fragments were then washed three times with phosphate-buffered saline (PBS) containing penicillin (100 U/mL) and streptomycin (100 μg/mL) (Sevenbio SC118). Sequential enzymatic digestion was performed: first with 0.25% (w/v) trypsin (Beyotime C0201) for 30 min at 37 °C, followed by digestion with 0.2% (w/v) type II collagenase (Biosharp BS164) for 3 hours at 37 °C. After digestion, the cell suspension was filtered through a sterile cell strainer to remove undigested tissue. The digested chondrocytes were seeded into a T25 culture flask.

### Cell culture and transfection

Primary chondrocytes were cultured in complete DMEM (Thermofisher 11965092) supplemented with 10% fetal bovine serum (FBS) (Absin abs972) and 1% penicillin-streptomycin (Sevenbio SC118). Chondrocytes at passages 3 to 5 were used for subsequent experiments. The NR8383 rat alveolar macrophage cell line was obtained from Cellverse. NR8383 cells were maintained in complete F-12K medium (Boster PYG0036) containing 15% FBS, 1% penicillin-streptomycin, and 1% L-glutamine (Cellverse iCell-0900). To induce M1-type polarization and simulate an *in vitro* KOA inflammatory environment, NR8383 macrophages were stimulated with lipopolysaccharide (LPS, 1 µg/mL) (Solarbio L8880) for 24 hours. After LPS stimulus, M1-type macrophages were treated with 10% PRP. Let-7a-5p mimic (Ribobio miR10000774-1-5), negative control (NC) (Ribobio miR1N0000001-1-5) and si-MAPK8 (purchased from Wuhan Lingsi Biotechnology Co., Ltd.) were transfected into NR8383 cells using lipofectamine 3000 (Thermo Fisher Scientific L3000001) following the manufacturer’s protocols.

### Preparation of RNA and quantitative RT-PCR

Total RNA was extracted using TRIzol reagent (Sevenbio SM129). Complementary DNA (cDNA) was synthesized from the extracted RNA using a commercial reverse transcription kit (Sevenbio SM134). Reverse transcription-quantitative polymerase chain reaction (RT-qPCR) was then performed using SYBR Green chemistry (Sevenbio SM143). All primers were synthesized by Sevenbio. Primer sequences are detailed in the Supplementary File (**Supplementary Table S1**). Gene expression levels of target genes were normalized to *β*-actin and analyzed using the 2^−ΔΔCt^ method.

### Western blot

Cells grown in T25 flasks were lysed in 120 μl RIPA buffer supplemented (Beyotime P0013B) with protease inhibitors (Beyotime ST505). Protein concentration was measured using a commercial assay kit (Beyotime P0010). Equal amounts of protein were separated by SDS-PAGE (Epizyme PG112) and transferred to a PVDF membrane (Millipore IPVH00010). Membranes were blocked with rapid blocking buffer (Epizyme PS108P) for 15 min at room temperature and then incubated overnight at 4 °C with primary antibodies diluted in universal antibody dilution buffer (Sevenbio SW161). After three washes with TBST (Sevenbio SW142), membranes were incubated with horseradish peroxidase (HRP)-conjugated secondary antibody (Immunoway RS0002) for 1 hour at room temperature. Following three additional TBST washes, protein bands were visualized using enhanced chemiluminescence (ECL) detection (Meilune MA0186). The antibodies used were: anti-COL II (Immunoway YT1022), anti-ACAN (Immunoway YC0042), anti-MMP13 (Immunoway YT2796), anti-MAPK8 (Immunoway YM8450) and anti-GAPDH (Abway AB0037).

### Histological staining analysis

After receiving their respective treatments, the rats were euthanized, and knee joint samples were collected for histological examination. The samples were fixed in 4% paraformaldehyde (Sevenbio Sl101-01), and bone tissue was subsequently decalcified using EDTA (Sinopharm 10009717). Following decalcification, the tissues were dehydrated and embedded in paraffin. Sections of 4 μm thickness were prepared from the paraffin blocks and stained with HE (lingsibio E8090, G1140) as well as safranin O-fast green (Servicebio G1053). Synovitis and cartilage degeneration were evaluated using synovitis scores and the OARSI scoring system, respectively. For immunohistochemical (IHC) staining, paraffin-embedded sections were deparaffinized, rehydrated, and subjected to antigen retrieval and blocking. The sections were then incubated overnight at 4°C with primary antibodies against inducible nitric oxide synthase (iNOS) (Immunoway YT3169) and CD206 (wanleibio WL06177), followed by incubation with a secondary antibody (Ljsbio LJS-S-0001) for 30 min at 37°C. Finally, color development was performed using DAB (Servicebio G1212-200T), and the sections were counterstained with hematoxylin (Sigma H9627) and analyzed under a light microscope.

### Immunofluorescence staining

The tissue sections underwent a process of deparaffinized, rehydrated, and subjected to antigen retrieval and blocking. Cells were fixed with 4% paraformaldehyde (Sevenbio Sl101-01), permeabilized with 0.1% Triton X-100 (Biofroxx 1139), and blocked with 4% BSA (Sevenbio SO110). Subsequently, the tissue sections and cells were incubated overnight at 4°C with primary antibodies against iNOS (Proteintech 22226-1-AP), CD206 (Proteintech 18704-1-AP) and MAPK8 (Proteintech 66210-1-Ig). Following this, the tissue sections and cells were stained with the Goat anti-Rabbit IgG (H+L) (Thermo Fisher Scientific, A10520) or Goat anti-Mouse IgG (H+L) (Thermo Fisher Scientific, F2761) secondary antibodies, as well as DAPI (Sevenbio, SI111), in the dark. Finally, fluorescence images were captured using a fluorescence microscope.

### Bioinformatics

The dataset GSE200251 was retrieved from the GEO database using the keyword “platelet-rich plasma”. This dataset comprises high-throughput sequencing results of microRNAs from both PRP and platelet-poor plasma (PPP) obtained from four healthy donors. Differential expression analysis of microRNAs was performed using the R package “DESeq2”, with criteria set at |log_2_FoldChange| ≥ 1 and an adjusted *P* value < 0.05. Target genes of the differentially expressed microRNAs were identified through two complementary approaches: one utilizing the R package “multiMiR” to extract both predicted and experimentally validated targets, and the other integrating information from the CTD, DisGeNET, and GeneCards databases. Gene Ontology (GO) and Kyoto Encyclopedia of Genes and Genomes (KEGG) enrichment analyses were performed with the ClusterProfiler R package.

### Dual-luciferase reporter assay

In order to confirm the relationship between let-7a-5p and MAPK8, the wild-type or mutant 3′UTR of MAPK8 (WT-MAPK8, MUT-MAPK8) was constructed into dual luciferase reporters using pmirGLO vector. Then, the reporter vectors containing WT-MAPK8 or MUT-MAPK8 with let-7a-5p-mimic or let-7a-5p-inhibitor were co-transfected into NR8383 using Lipofectamine 3000 (Thermo Fisher Scientific L3000001). The luciferase activities of renilla and firefly were measured using a dual luciferase reporter assay kit (Beyotime RG027) after 48h.

### Statistical analysis

Statistical analysis and data visualization were conducted using GraphPad Prism 9.5.1 software. Data are expressed as mean ± standard deviation (SD). Comparisons between two groups were made using an unpaired *t*-test, while comparisons among multiple groups were performed using one-way ANOVA. A *P* value < 0.05 was considered statistically significant.

## Results

### PRP alleviates cartilage degeneration and synovial inflammation in the KOA rat model

**Figure 1:**
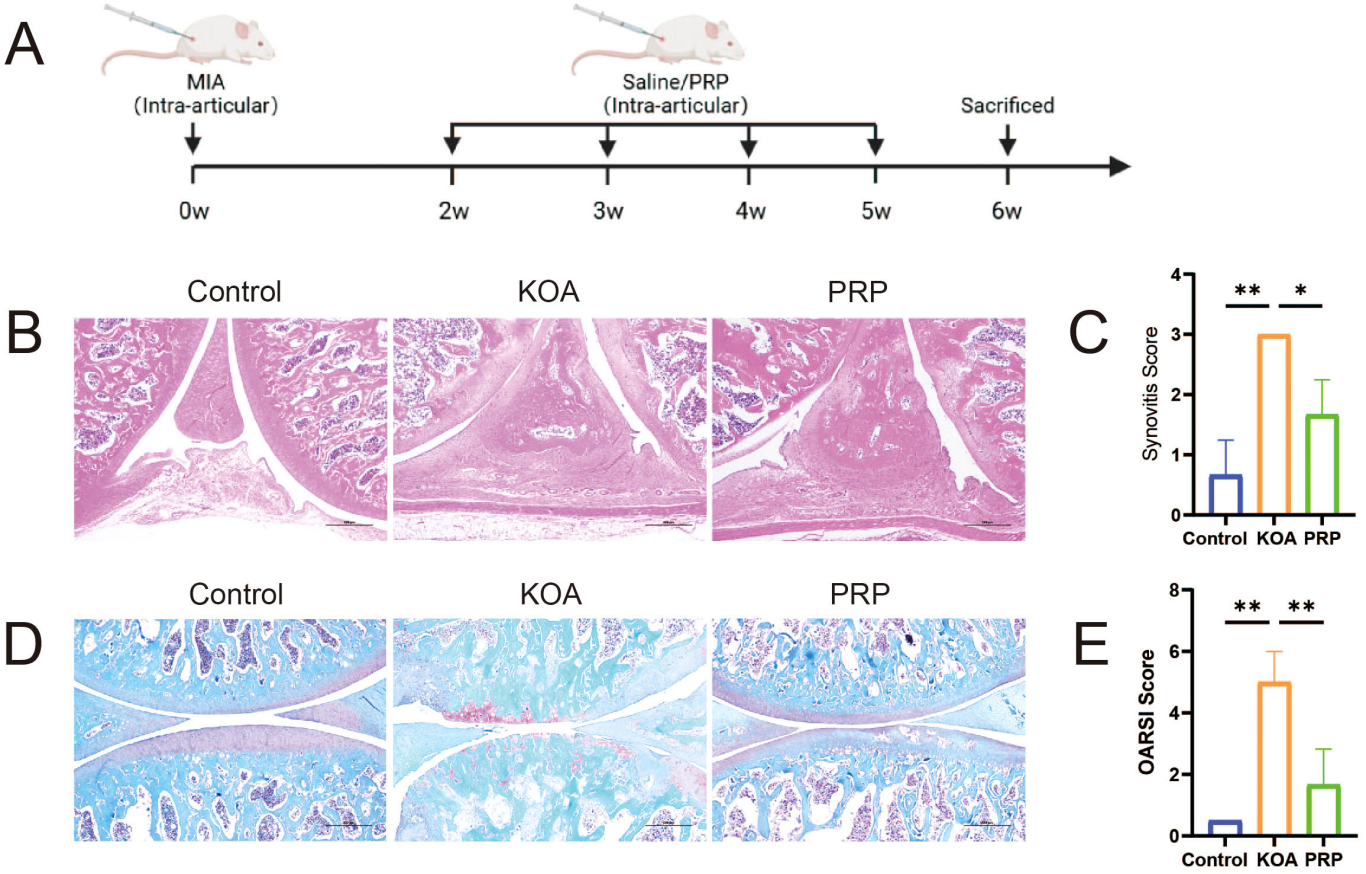
Efficacy of PRP in alleviating cartilage degeneration and synovial inflammation in a KOA rat model. **(A)** Schematic illustration of the experimental design in SD rat with KOA. **(B)** HE staining of synovial tissues in each group (Scale bar: 500 μm). **(C)** Quantification of synovitis score based on HE staining (n = 3). **(D)** Safranin O-fast green staining of knee joint articular cartilage in each group (Scale bar: 500 μm). **(E)** Quantification of OARSI grades based on Safranin O-fast green staining (n = 3). (*P < 0.05, **P < 0.01, ***P < 0.001).

The KOA rat model was successfully established using MIA induction (**Figure 1A**). Synovial inflammation severity was assessed by examining rat knee joints with HE staining, revealing more pronounced inflammatory cell infiltration in the KOA group compared to the Control group (**Figures 1B, C**, *P* < 0.01). This infiltration was reduced following PRP treatment (**Figures 1B, C**, *P* < 0.05). Evaluation of cartilage degeneration was conducted by analyzing rat knee joint articular cartilage with Safranin O-fast green staining, indicating diminished Safranin O staining and noticeable cartilage degeneration in the KOA group relative to the control group. Conversely, the PRP group demonstrated marked improvement in the knee joints. Furthermore, OARSI scoring was performed, confirming significantly higher scores in the KOA group compared to both the control and PRP groups (**Figures 1D, E**, *P* < 0.01). Taken together, these findings suggest that PRP treatment can ameliorate both cartilage degeneration and synovial inflammation in KOA rats.

### PRP modulates macrophage polarization improve the inflammatory microenvironment

**Figure 2:**
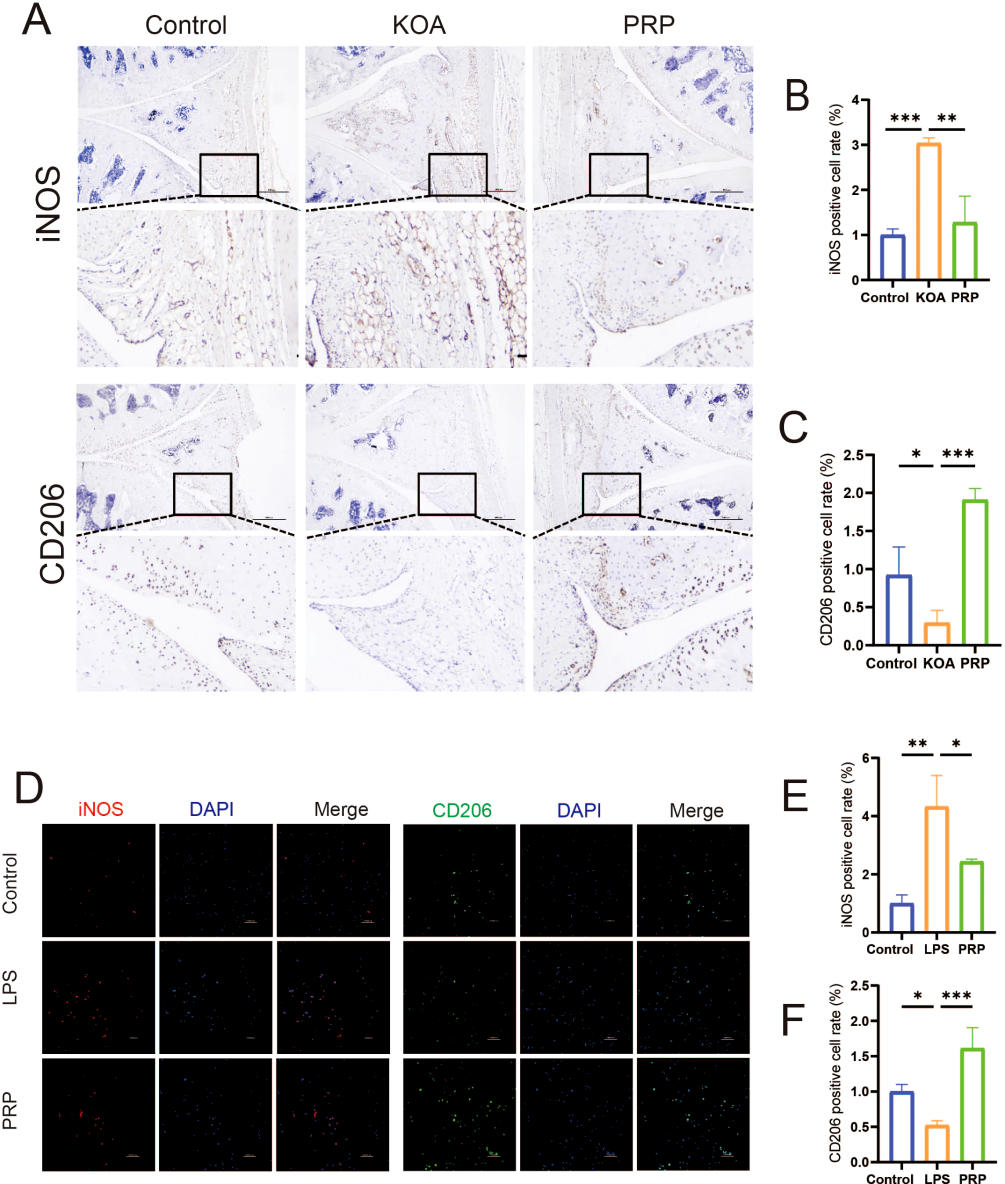
Effect of PRP on modulating macrophage polarization. **(A)** IHC staining for M1 marker iNOS and M2 marker CD206 in synovial tissue sections of each group (Scale bar: 500 μm). **(B)** Quantification of iNOS-positive cell rate in synovial tissue (n = 3). **(C)** Quantification of CD206-positive cell rate in synovial tissue (n = 3). **(D)** IF staining showing expression of iNOS and CD206 in macrophages *in vitro* (Scale bar: 100 μm). **(E)** Quantification of iNOS-positive cell rate *in vitro*. **(F)** Quantification of CD206-positive cell rate *in vitro*. (*P < 0.05, **P < 0.01, ***P < 0.001).

To investigate the effect of PRP on the inflammatory microenvironment in KOA, we established *in vivo* (MIA-induced) and *in vitro* (LPS-induced) KOA models (**Supplementary Figures S1, 2**). IHC staining and IF were used to assess the effects of PRP on macrophage polarization towards M1/M2 phenotypes by detecting the iNOS and CD206 expression both *in vivo* and *in vitro*. IHC analysis revealed that *in vivo* (**Figures 2A–C**), the proportion of iNOS-positive cells was higher in the KOA group compared to controls (*P* < 0.001), while PRP treatment significantly reduced the proportion of iNOS-positive cells (*P* < 0.01). Conversely, the number of CD206-positive cells was lower in the KOA group (*P* < 0.05), and PRP treatment increased the number of CD206-positive cells(*P* < 0.001). Similarly, immunofluorescence results demonstrated that *in vitro* (**Figures 2D–F**), PRP treatment suppressed M1 phenotype macrophages and enhanced M2 phenotype macrophages.

**Figure 3:**
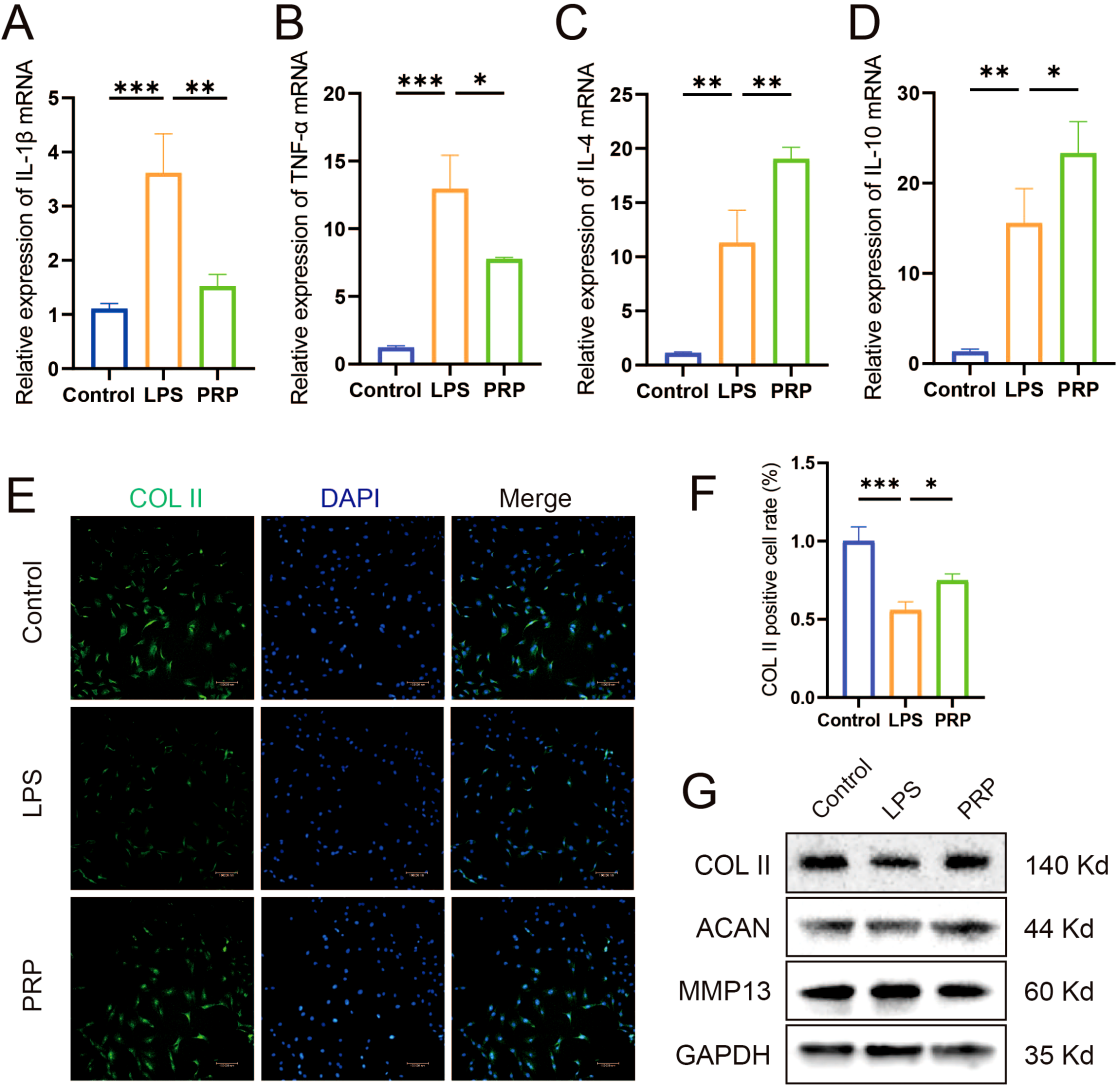
Effect of PRP on improving the inflammatory microenvironment. **(A-D)** Relative mRNA expression levels of pro-inflammatory cytokine (IL-1β and TNF-α) and anti-inflammatory cytokine (IL-4 and IL-10) in macrophages detected by RT-qPCR. **(E)** IF staining showing expression of COL II in chondrocytes treated with macrophage conditioned medium (Scale bar: 100 μm). **(F)** Quantification of COL II positive cell rate in chondrocytes. **(G)** WB analysis of COL II, ACAN, and MMP13 protein expression in chondrocytes treated with macrophage CM. (*P < 0.05, **P < 0.01, ***P < 0.001).

Macrophage polarization critically influences the inflammatory microenvironment of the knee joint. M1-type polarization releases pro-inflammatory cytokines, whereas M2-type polarization releases anti-inflammatory cytokines. qRT-PCR was used to detect the release of pro-inflammatory and anti-inflammatory cytokine. As shown in **Figures 3A–D**, compared with the LPS group, the expression levels were significantly downregulated of pro-inflammatory cytokine (IL-1β and TNF-α) and upregulated of anti-inflammatory cytokine (IL-4 and IL-10) in LPS-induced macrophages intervened by PRP. Similarly, PRP suppresses pro-inflammatory cytokine secretion *in vivo* (**Supplementary Figure S3**).

To investigate the interaction between macrophages and chondrocytes, conditioned media (CM) were collected from macrophage cultures following various treatments to simulate the macrophage-derived inflammatory microenvironment and explore potential crosstalk between these cells. Chondrocyte degeneration was then assessed by evaluating extracellular matrix (ECM) markers. IF results demonstrated that, compared to the control group, chondrocytes treated with LPS-CM exhibited decreased expression of COL II (**Figures 3E, F**, *P < 0.05*). WB results demonstrated that, compared to the control group, chondrocytes treated with LPS-CM exhibited decreased expression of COL II and ACAN, alongside increased MMP13 expression (**Figure 3G**). Importantly, PRP-CM effectively reversed these alterations. Collectively, these results indicate that PRP ameliorates the inflammatory microenvironment in knee osteoarthritis by modulating macrophage polarization, thereby reducing chondrocyte degeneration under inflammatory conditions.

### Differential expression of miRNAs in PRP

**Figure 4:**
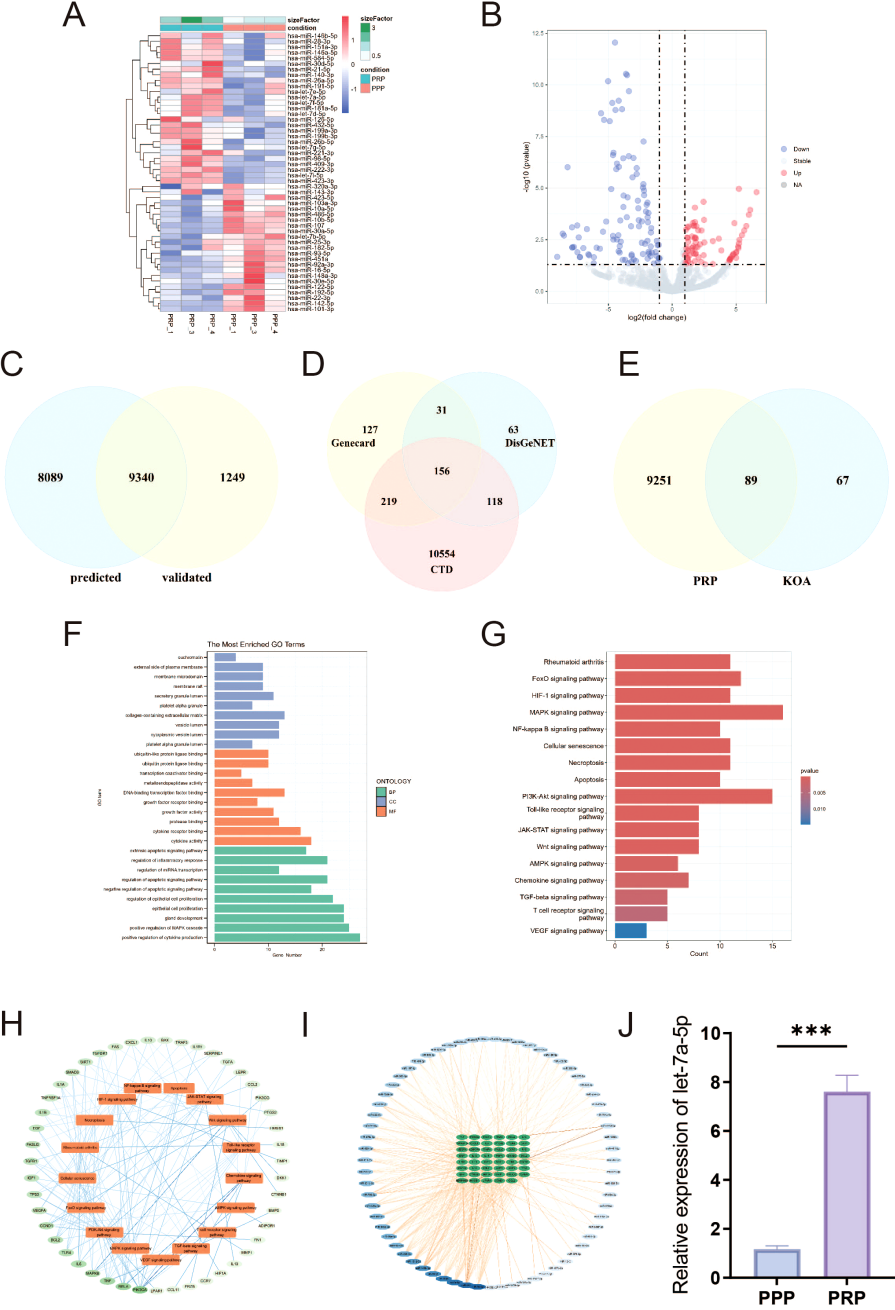
Bioinformatics identification of differentially expressed miRNAs in PRP. **(A, B)** DEGs between PPP and PRP group were presented by heatmap and volcano plot. **(C)** Venn diagram illustrating the intersection of predicted and validated target genes. **(D)** Venn diagram showing intersection of KOA-associated genes identified from CTD, DisGeNET, and GeneCards databases. **(E)** Venn diagram presenting the intersection of miRNAs target genes and KOA-associated genes. **(F)** GO enrichment analysis of target genes. **(G)** KEGG enrichment analysis of target genes. **(H, I)** Screening process for advantageous pathways and microRNA. **(J)** Relative expression level of let-7a-5p in PPP and PRP detected by RT-qPCR. (***P < 0.001).

To determine whether PRP-derived microRNAs mediate the amelioration of the inflammatory microenvironment in KOA, we conducted bioinformatics analysis on the dataset GSE200251 from the GEO database to identify miRNAs differentially expressed in PRP. The results showed that there were 186 differentially expressed miRNAs, including 80 upregulated miRNAs and 106 downregulated miRNAs (|log_2_FoldChange| ≥ 1, *P* < 0.05), (**Figures 4A, B**) (**Supplementary Table S1**). Using the R package “multiMiR”, we analyzed both predicted and validated microRNA target genes. The common targets from these two datasets were then identified as the final set of microRNA targets (**Figure 4C**; **Supplementary Table S2**). Furthermore, we identified genes associated with KOA by querying the CTD, DisGeNET, and GeneCards databases (**Figure 4D**; **Supplementary Table S3**). Intersection analysis of these two gene sets revealed key target genes potentially mediating PRP’s effects on KOA (**Figure 4E**; **Supplementary Table S4**). Subsequently, GO (**Figure 4F**) and KEGG (**Figure 4G**) enrichment analyses were performed to identify key pathways. Pathways relevant to KOA pathogenesis were selected, leading to a final list of key target genes (**Figure 4H**). Finally, by analyzing miRNAs linked to these key targets (**Figure 4I**), we selected let-7a-5p for experimental evaluation. RT-qPCR analysis confirmed that let-7a-5p expression was significantly elevated in PRP compared to PPP (**Figure 4J**, *P* < 0.001). Subsequently, we will focus on elucidating the biological roles of let-7a-5p in mediating the amelioration of the inflammatory microenvironment in KOA.

### Let-7a-5p modulates macrophage polarization improve the inflammatory microenvironment

**Figure 5:**
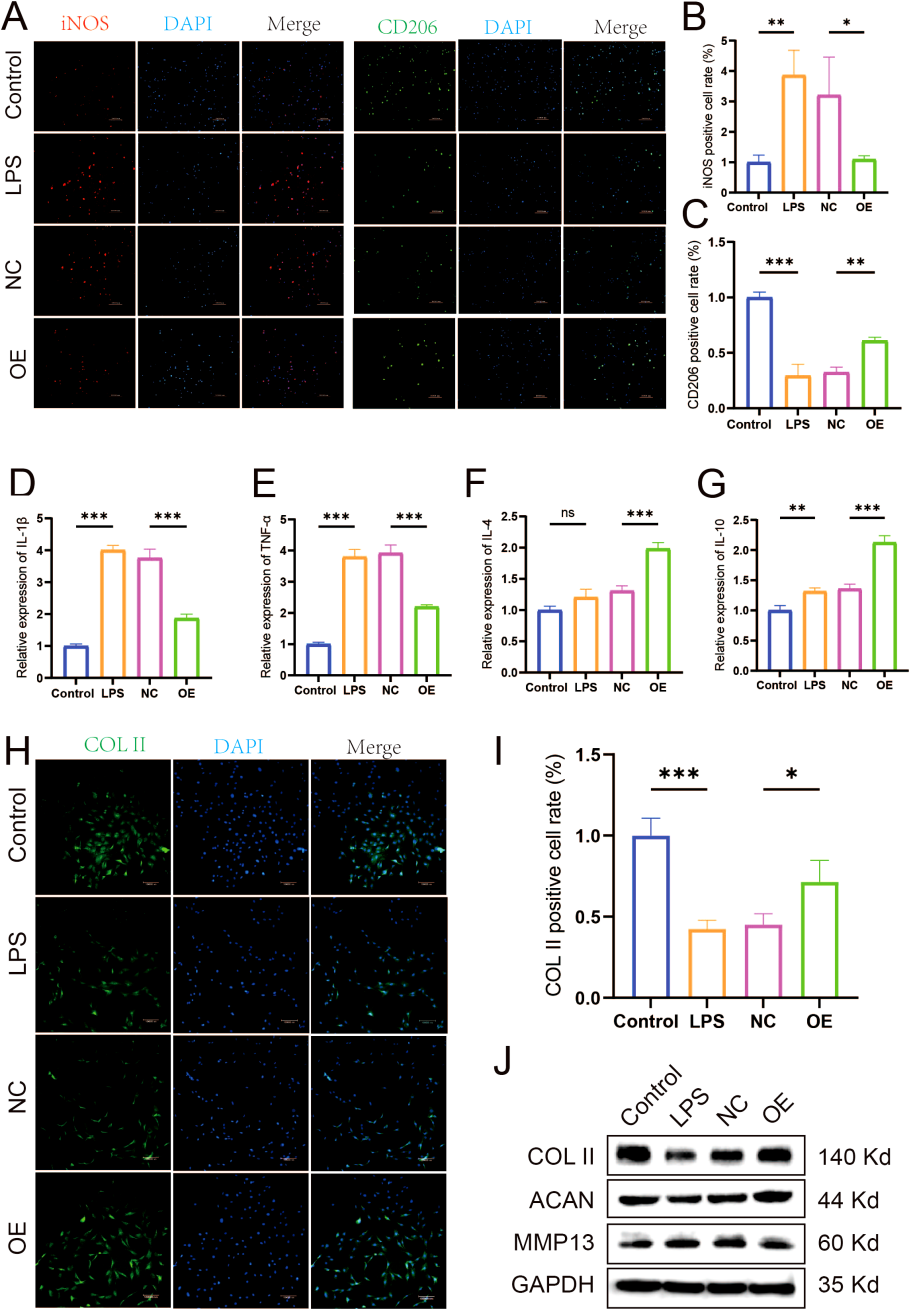
Role of Let-7a-5p in modulating macrophage polarization and improving the inflammatory microenvironment. **(A)** IF staining showing expression of iNOS and CD206 in macrophages transfected with let-7a-5p mimic (OE) or negative control (NC) (Scale bar: 100 μm). **(B)** Quantification of iNOS positive cell rate. **(C)** Quantification of CD206 positive cell rate. **(D-G)** Relative mRNA expression levels of pro-inflammatory cytokine (IL-1β and TNF-α) and anti-inflammatory cytokine (IL-4 and IL-10) in transfected macrophages detected by RT-qPCR. **(H)** IF staining showing expression of COL II in chondrocytes treated with conditioned medium from transfected macrophages (Scale bar: 100 μm). **(I)** Quantification of COL II positive cell rate in chondrocytes. **(J)** WB analysis of COL II, ACAN, and MMP13 protein expression in chondrocytes treated with conditioned medium from transfected macrophages. (*P < 0.05, **P < 0.01, ***P < 0.001).

To explore the specific biological role of let-7a-5p in KOA pathogenesis, we investigated its involvement in PRP-mediated amelioration of the inflammatory microenvironment in KOA. We transfected LPS-induced M1-type polarized macrophages with a let-7a-5p mimic (**Supplementary Figure S4**). Subsequently, the effects of let-7a-5p overexpression on macrophage polarization and inflammatory cytokine release were assessed. IF analysis revealed that, compared to the NC group, transfection with the let-7a-5p mimic reversed the expression of the M1 polarization marker iNOS and the M2 polarization marker CD206 in macrophages (**Figures 5A–C**). Similarly, RT-qPCR results showed that the let-7a-5p mimic significantly downregulated pro-inflammatory cytokine (IL-1β and TNF-α) expression and upregulated anti-inflammatory cytokine (IL-4 and IL-10) expression (**Figures 5D–G**, *P* < 0.001). Subsequently, the effect of let-7a-5p conditioned medium (let-7a-5p CM) on chondrocyte degeneration was assessed by analyzing indicators of cartilage degeneration. IF results demonstrated that, compared to the NC group, let-7a-5p CM significantly upregulated COL II expression (**Figures 5H, I**, *P* < 0.05). WB results demonstrated that, compared to the NC group, let-7a-5p CM significantly upregulated COL II and ACAN expression while downregulating MMP13 expression (**Figure 5J**). Collectively, let-7a-5p is essential for the biological effects of PRP by regulating macrophage polarization, which significantly reduces pro-inflammatory cytokine and thereby alleviates chondrocyte degeneration.

### MAPK8 is a direct functional target of let-7a-5p

**Figure 6:**
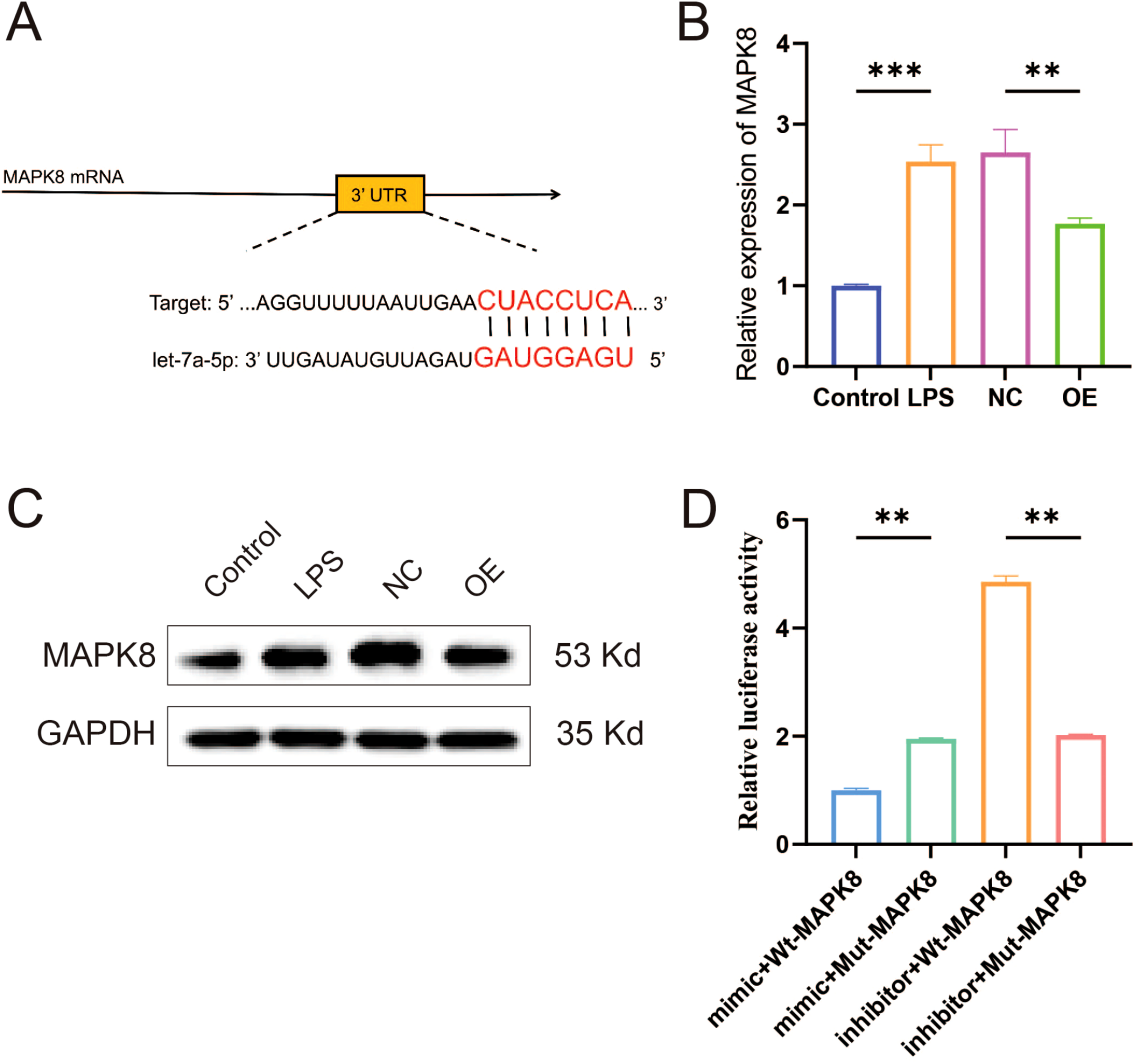
Validation of the targeting relationship between let-7a-5p and MAPK8. **(A)** Schematic representation of the predicted complementary binding site between let-7a-5p and the 3′-UTR of MAPK8. **(B)** Relative mRNA expression level of MAPK8 in macrophages overexpressing let-7a-5p detected by RT-qPCR. **(C)** WB analysis of MAPK8 protein expression in macrophages overexpressing let-7a-5p. **(D)** Inhibitory effect of let-7a-5p on MAPK8 expression was assessed via a dual luciferase reporter assay. (**P < 0.01, ***P < 0.001).

To elucidate the mechanism underlying let-7a-5p-mediated amelioration of the inflammatory microenvironment in KOA, we employed TargetScan to predict potential binding sites. This analysis identified a conserved let-7a-5p binding site within the 3′-UTR of the MAPK8 gene (**Figure 6A**). Subsequently, RT-qPCR and WB analyses confirmed a significant reduction in MAPK8 expression in macrophages overexpressing let-7a-5p (**Figures 6B, C**, *P* < 0.01). Dual-luciferase reporter assay revealed that the luciferase activity was significantly suppressed by let-7a-5p mimics in NR8383 cells co-transfected with WT-MAPK8 luciferase reporter plasmid. In contrast, no significant change was observed when co-transfected with MUT-MAPK8 plasmid and let-7a-5p mimics (**Figure 6D**, *P* < 0.05). These results demonstrate that MAPK8 is a direct target gene of let-7a-5p.

### MAPK8 regulates macrophage polarization to improve the inflammatory microenvironment

**Figure 7:**
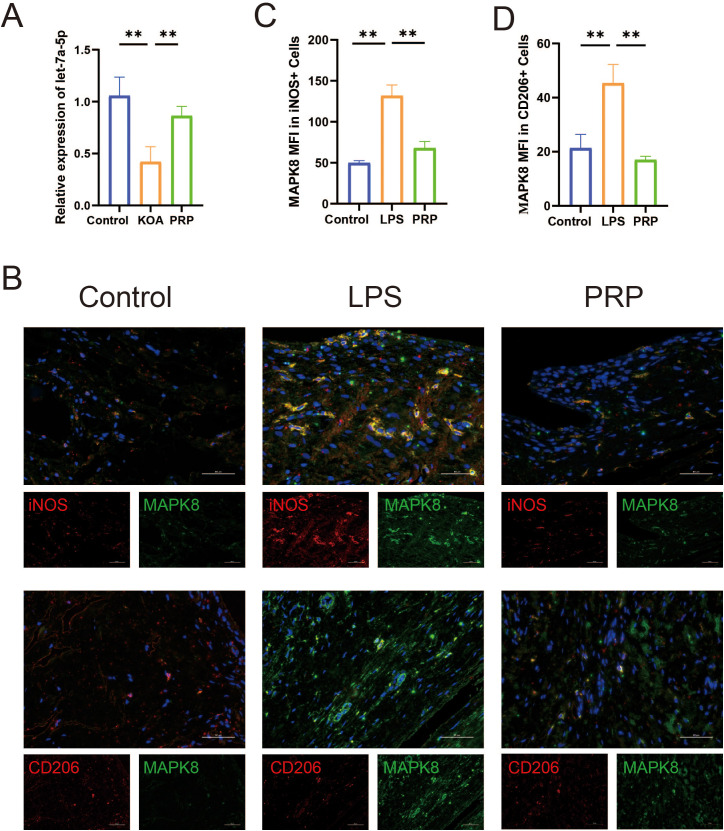
Effect of PRP on expression of let-7a-5p and MAPK8. **(A)** Relative mRNA expression levels of let-7a-5p in knee joint sections of each group detected by RT-qPCR. **(B)** iNOS and CD206 co-immunolabeld with MAPK8 and counter-stained with DAPI in synovial tissues (Scale bar: 50 μm). **(C, D)** Quantification of iNOS and CD206 expression co-localized with MAPK8. (**P < 0.01).

To further investigate the molecular mechanism by which MAPK8 axis regulates macrophage polarization both *in vivo* and *in vitro*, We first examined the expression of let-7a-5p *in vivo*. RT-qPCR results showed that let-7a-5p expression was significantly upregulated in the PRP group compared with the KOA model group (**Figure 7A**, *P* < 0.05). Subsequently, we verified the involvement of MAPK8 in macrophage polarization *in vivo.* Immunofluorescence co-staining with MAPK8 revealed an increase in iNOS and a decrease in CD206 in the KOA model group. However, these changes were reversed by PRP treatment (**Figures 7B-D**, *P* < 0.01).

**Figure 8:**
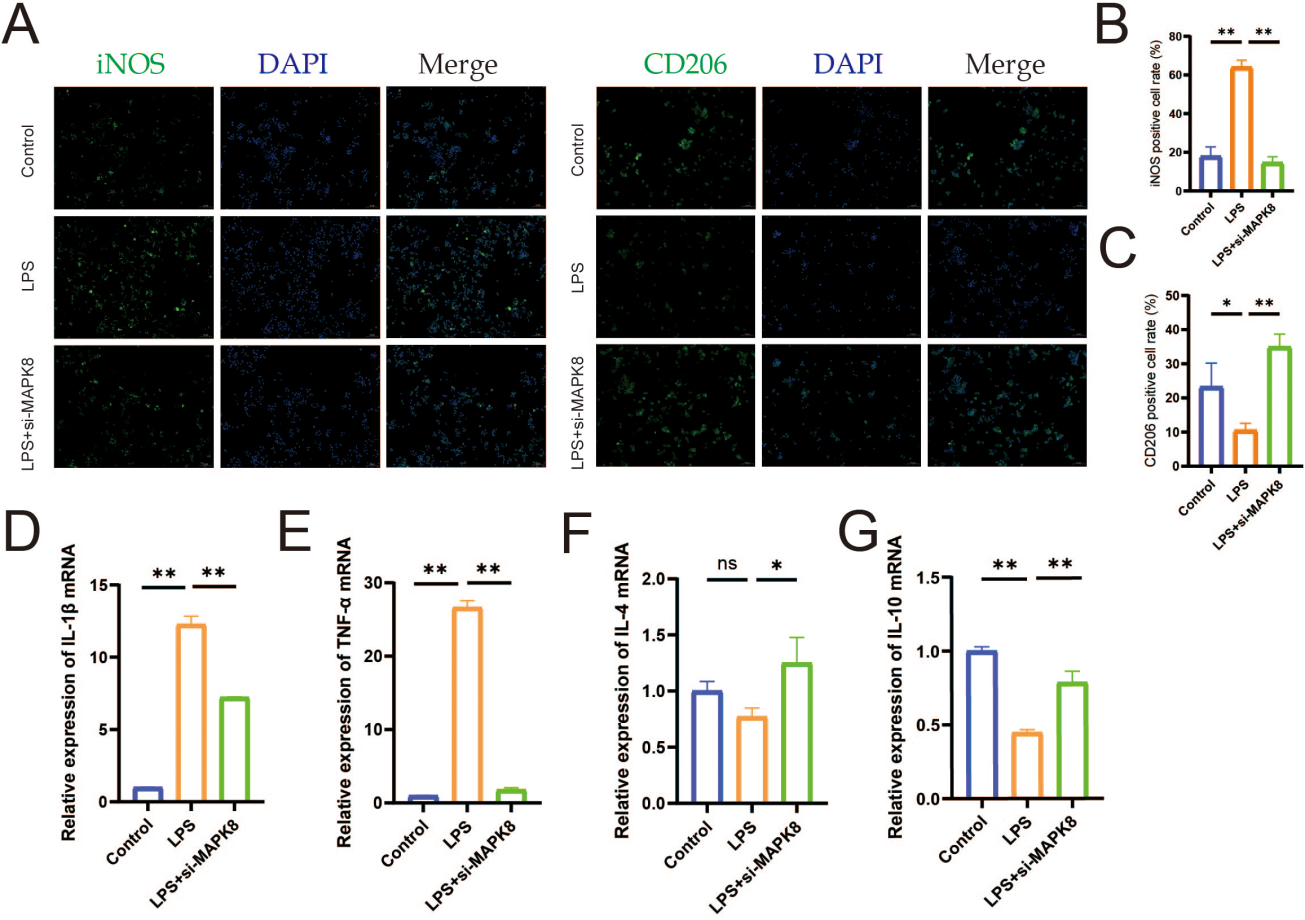
The let-7a-5p/MAPK8 axis regulates macrophage polarization and inflammatory cytokine release *in vitro*. **(A)** IF staining showing expression of iNOS and CD206 in macrophages (Scale bar: 50 μm). **(B)** Quantification of iNOS-positive cell rate in transfected macrophages. **(C)** Quantification of CD206-positive cell rate in transfected macrophages. **(D-G)** Relative mRNA expression levels of pro-inflammatory cytokine (IL-1β and TNF-α) and anti-inflammatory cytokine (IL-4 and IL-10) in transfected macrophages detected by RT-qPCR. (*P < 0.05, **P < 0.01, ns no significance).

We conducted *in vitro* validation of the molecular mechanism of MAPK8 in LPS-induced macrophages. Firstly, we downregulated MAPK8 expression in LPS-induced macrophages (**Supplementary Figure S5**) and IF results revealed that compared to the LPS group, transfection with si-MAPK8 reversed the expression of the M1 polarization marker iNOS and the M2 polarization marker CD206 in macrophages (**Figures 8A–C**, *P* < 0.01). Then, RT-qPCR analysis revealed that the mRNA levels of IL-1β and TNF-α were increased, whereas those of IL-4 and IL-10 were decreased in the LPS group. However, inhibition of MAPK8 reversed this imbalance in inflammatory cytokine expression (**Figures 8D–G**, *P* < 0.01). These data indicated that MAPK8 is indispensable for let-7a-5p-mediated macrophage polarization.

## Discussion

Knee osteoarthritis is very common among the older adults, which can cause joint stiffness, pain, and even disability, imposing a huge burden on families and society, and is one of the main causes of pain and disability worldwide (20, 21). KOA is regarded as the mechanical “wear and tear” of articular cartilage, and recent studies have widely confirmed the critical role of inflammation. The inflammatory microenvironment is now considered closely related to the occurrence and progression of KOA (4). Traditional non-surgical treatment methods for KOA can only alleviate symptoms and are not conducive to repair, while PRP injection therapy has been widely recognized by patients and researchers for its ability to alleviate symptoms and repair joint cartilage (9, 10). Previous studies demonstrate that intra-articular PRP injection promotes cartilage repair (22) and reduces synovial inflammation (23) in KOA rat models. Our research results also show that PRP can alleviate the degree of cartilage degeneration and synovitis in rats with KOA. HE staining and Safranin O-fast green staining showed that compared with the KOA group, the degree of knee joint synovitis and cartilage degeneration were improved in the PRP group.

Synovial macrophages are a mixed population divided into M1 and M2 phenotypes according to different functional phenotypes (24). When synovitis occurs in the knee joint, synovial macrophages undergo M1-type polarization, releasing inflammatory cytokine to disrupt the balance between extracellular matrix anabolism and catabolism, leading to cartilage degeneration. The degenerated cartilage further stimulates M1-type macrophage polarization, aggravating synovitis and establishing a vicious cycle (25). In this study, we found that the proportion of iNOS-positive cells in the KOA group was significantly greater than that in the control group, while PRP treatment significantly reduced the proportion of iNOS-positive cells. Conversely, the proportion of CD206-positive cells was lower in the KOA group, and PRP intervention increased the proportion of CD206-positive cells. Xu et al. (26) found that on days 5 and 21, the proportion of iNOS-positive cells in the MIA group was significantly greater than that in the sham group, indicating that the proportion of M1/M2 macrophages increased and persisted throughout KOA. Moreover, the development of novel probes such as CD206 and formyl peptide receptor 1 targeting other macrophage markers has also confirmed that OA can be treated by altering macrophage phenotype (27, 28). Our results showed that synovial macrophages in the KOA group were polarized into M1 phenotype, while macrophages transformed into M2 phenotype after PRP intervention, which is consistent with previous studies (29, 30). This also indicates that the mechanism of PRP treatment for KOA is related to its regulation of macrophage polarization.

Consistent with previous studies, our results also indicate that PRP can significantly reduce the levels of IL-1β and TNF-α secreted by M1 macrophages, and increase the levels of IL-4 and IL-10 released by M2 macrophages. And compared with the control group, the expression of type II collagen in chondrocytes treated with LPS-CM was reduced, and PRP intervention effectively reversed this change. Given the central role of macrophages in driving synovial inflammation (8), while IL-1β and TNF-α are key pro-inflammatory cytokines that exert major destructive effects within the inflammatory microenvironment (31). Existing studies demonstrate that modulating macrophage M1-type polarization often directly influences the release of inflammatory cytokine (32). In addition, the excessive secretion of inflammatory cytokine such as IL-1β, IL-6, and TNF-α can also stimulate destructive processes in chondrocytes, such as downregulating the synthesis of type II collagen and aggrecan, limiting cartilage formation (33). We were surprised to find a report indicating that an increase in type II collagen (SCII) in squid can be achieved by increasing the proportion of M2 macrophages and chondrogenic cytokines (TGF-b1 and TGF-b3) in synovial fluid (34). Collectively, these results robustly confirm that PRP ameliorates the inflammatory microenvironment of KOA by regulating macrophage polarization and the subsequent balance of pro-/anti-inflammatory cytokines.

Accumulating evidence indicates that specific miRNAs play either protective or detrimental roles in the development and progression of KOA (35). However, studies focusing on PRP-related miRNAs regulating macrophage polarization for KOA intervention are still lacking. There are reports that miRNAs highly expressed in OA are associated with macrophage polarization. MiR-155-5p has been previously found to associate with OA, where increased miRNA-155-5p expression leads to the M1 polarization of the RAW 264.7 macrophage cell line (36). Li et al. indicated that the proinflammatory microenvironment of KOA synovial fluid may favor M1 macrophage polarization by upregulating miR-155-5p whilst inhibiting M1 macrophage apoptosis (37). A study has shown that miR-145 can protect chondrocytes from degradation and its content is significantly reduced in KOA chondrocytes. Transfection of miR-145 significantly enhanced chondrocyte proliferation and attenuated ECM degradation (38). Research has shown that some miRNAs contribute to the development and progression of OA by regulating the inflammatory process, or by affecting the balance between cartilage and affecting cartilage metabolism, synthesis metabolism, and degradation metabolism within chondrocytes. In addition, some miRNAs can also affect chondrocyte apoptosis, which contributes to the degradation of OA cartilage. In addition to its effect on chondrocytes, miRNA also exerts its function by regulating osteoarthritis synovial fibroblasts (39, 40). After database screening analysis and validation, let-7a-5p, which is significantly enriched in PRP compared to PPP, was identified as a candidate miRNA. Although the role and mechanism of let-7a-5p in other diseases have been confirmed, such as alleviating mouse pulmonary fibrosis by inhibiting pyroptosis and inflammation (41), and promoting chondrocyte migration and proliferation in osteoarthritis (42), there have been no reports on its application in PRP treatment of KOA. Our results revealed that after transfection with let-7a-5p mimic, the M1 polarization marker iNOS decreased accompanied by the expression downregulation of pro-inflammatory cytokine (IL-1β and TNF-α) expression, while the M2 polarization marker CD206 and the expression of anti-inflammatory cytokine (IL-4 and IL-10) increased. Furthermore, the expression of COL II was significantly upregulated compared with the NC group, indicating that let-7a-5p can significantly protect chondrocytes from degradation. In summary, we have reason to believe that PRP can regulate macrophage polarization through its associated miRNA let-7a-5p, reverse the ratio of pro-inflammatory and anti-inflammatory cytokine, and alleviate chondrocyte degeneration.

Subsequently, we sought to further elucidate the molecular mechanism underlying the therapeutic effect of let-7a-5p in KOA. TargetScan analysis predicted MAPK8 as a potential target of let-7a-5p. Validating this prediction, our dual-luciferase reporter assay provided strong evidence that let-7a-5p negatively regulates MAPK8 expression. It is widely known that the MAPK family is closely involved in inflammation-related diseases, including KOA (43). MAPK8, as a key member of the MAPK family, also participates in a variety of physiological and pathological processes. Recent studies indicate that Bailixiang tea alleviates lung tissue damage via the TLR2/MAPK8 pathway (44), and miR-130a-3p attenuates endothelial inflammation by inhibiting MAPK8 (45). Consistent with these findings, we found that the downregulation of MAPK8 attenuated M1 macrophage polarization and the release of inflammatory cytokine. Collectively, these data suggest that let-7a-5p/MAPK8 axis is essential for modulating macrophage polarization and improving the inflammatory microenvironment.

While our study presents novel findings, several limitations should be acknowledged. First, PRP is a complex biological cocktail of numerous growth factors, cytokines, and miRNAs. Its overall therapeutic effect likely stems from the synergistic actions of multiple components. Thus, other molecules within PRP may also contribute to macrophage regulation, and the intricate interactions among them warrant further exploration. Second, although we confirmed via RT-qPCR that let-7a-5p is significantly enriched in PRP compared to PPP, we did not include a PPP intervention group *in vitro* experiments. We focused primarily on the specific molecular mechanism of the let-7a-5p/MAPK8 axis rather than a comprehensive comparison of different blood components. While our data strongly suggest that the therapeutic efficacy is driven by platelet-derived factors, future studies incorporating PPP and other blood fractions as parallel controls would be beneficial to further delineate the contribution of plasma-borne cytokines versus platelet-derived miRNAs in KOA treatment. Lastly, we have confirmed that MAPK8 is a direct target of let-7a-5p, but its functional relevance has not been validated through *in vivo* interventions of let-7a-5p or MAPK8. Subsequent research will focus on these *in vivo* regulatory mechanisms to provide clearer evidence for the therapeutic potential of the let-7a-5p/MAPK8 axis in KOA. Furthermore, this study opens promising avenues for KOA therapies, identifying let-7a-5p as a highly promising therapeutic target. The direct intra-articular delivery of let-7a-5p mimics, or their targeted delivery to synovial macrophages using nanoparticle-based systems, could pave the way for a novel and more precise biologic treatments for KOA.

PRP is very promising in the treatment of articular cartilage injury. The technology of PRP in the repair and treatment of articular cartilage injury is worthy of further research, which will benefit the majority of patients with KOA and articular cartilage injury.

## Conclusion

Our study demonstrates that let-7a-5p derived from PRP alleviates KOA by modulating the inflammatory microenvironment. Specifically, PRP-derived let-7a-5p effectively shifts macrophage polarization by inhibiting the pro-inflammatory M1 phenotype while promoting the anti-inflammatory M2 phenotype. This functional change significantly reduces the release of pro-inflammatory cytokines (IL-1β and TNF-α), thereby slowing cartilage degeneration. Mechanistically, we identified MAPK8 as a direct target gene of let-7a-5p, demonstrating that the inhibitory effect of the let-7a-5p/MAPK8 signaling axis is key to mediating these biological outcomes. Overall, within the complex inflammatory milieu of KOA, PRP regulated let-7a-5p is a promising target and offers novel therapeutic insights for disease management.

## Data Availability

Publicly available datasets were analyzed in this study. This data can be found here: GEO database: dataset GSE200251.
