# Supplementary material for: Platelet-rich plasma-derived microRNA let-7a-5p alleviates knee osteoarthritis by regulating macrophage polarization and improving inflammatory microenvironment
Source: Front Immunol. 2026 Feb 16;17:1756467. doi: 10.3389/fimmu.2026.1756467 (PMC12950549; doi:10.3389/fimmu.2026.1756467)
Supplement: Supplementary file 1 [file Supplementaryfile1.docx]

Supplementary Table 1. Sequences of primers used in real-time quantitative PCR.

| iNOS -R | TGGGTGTCAGAGTCTTGTGC |
| --- | --- |
| IL-1β-F | TACCTATGTCTTGCCCGTGG |
| IL-1β-R | TTTTGTCGTTGCTTGTCTCT |
| TNF-α-F | CCTCCAGAACTCCAGGCGGT |
| TNF-α-R | GCTTGGTGGTTTGCTACGAC |
| IL-4 -F | CAGGTCACAGAAAAAGGGAC |
| IL-4 -R | GAAGTAAAATTTGCGAAGCA |
| IL-10 -F | CAGACCCACATGCTCCGAGA |
| IL-10 -R | GCAACCCAAGTAACCCTTAA |
| let-7a-5p - F | GCGCGTGAGGTAGTAGGTTGT |
| let-7a-5p - R | AGTGCAGGGTCCGAGGTATT |
| MAPK-8-F | ACTGTTTCCTGATGTGCTTTT |
| MAPK-8-R | CTCCTCTATTGTGTGCTCCCT |
| β-actin -F | TGACGTTGACATCCGTAAAGACC |
| β-actin -R | GTGCTAGGAGCCAGGGCAGTAA |
| U6 - F | CGCTTCGGCAGCACATATAC |
| U6 - R | AAATATGGAACGCTTCACGA |

**Supplementary figure1:**


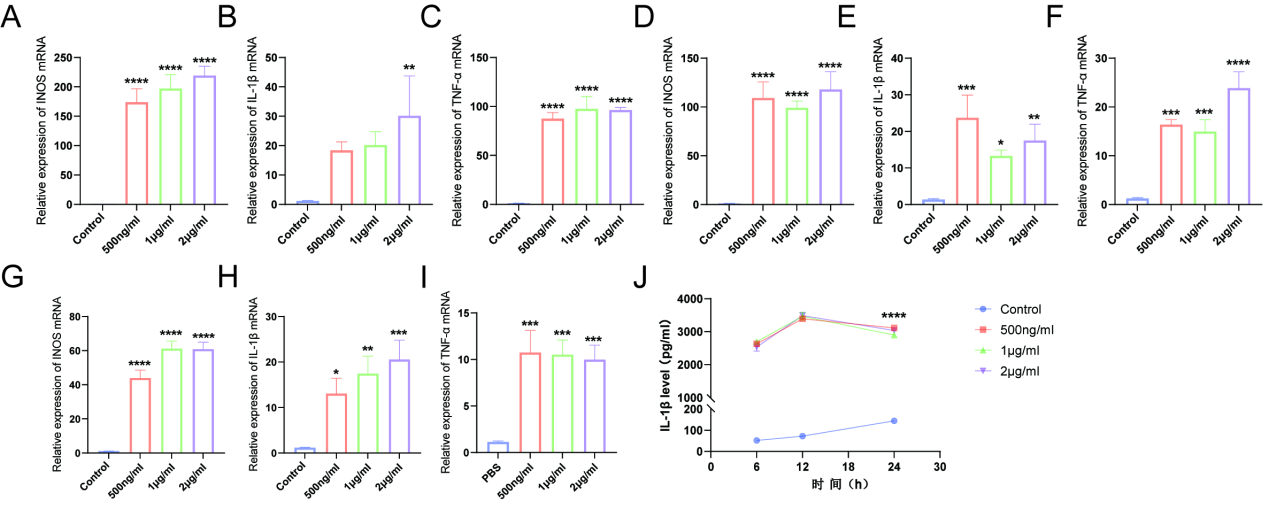


**Optimization of LPS-induced M1-type macrophage polarization.** (A-C) Relative mRNA expression levels of M1-type macrophage polarization marker iNOS and pro-inflammatory factors IL-1β and TNF-α after treatment with different concentrations of LPS (500 ng/ml, 1 µg/ml, and 2 µg/ml) for 6 hours. (D-F) Relative mRNA expression levels of M1-type macrophage polarization marker iNOS and pro-inflammatory factors IL-1β and TNF-α after treatment with different concentrations of LPS (500 ng/ml, 1 µg/ml, and 2 µg/ml) for 12 hours. (G-I) Relative mRNA expression levels of M1-type macrophage polarization marker iNOS and pro-inflammatory factors IL-1β and TNF-α after treatment with different concentrations of LPS (500 ng/ml, 1 µg/ml, and 2 µg/ml) for 24 hours. (J) ELISA analysis of IL-1β protein concentration in the supernatant after treatment with different concentrations of LPS (500 ng/ml, 1 µg/ml, and 2 µg/ml) for different time points (6 h, 12 h, and 24 h). (**P* < 0.05, ***P* < 0.01, ****P* < 0.001, *****P* < 0.0001)

**Supplementary figure2:**

**
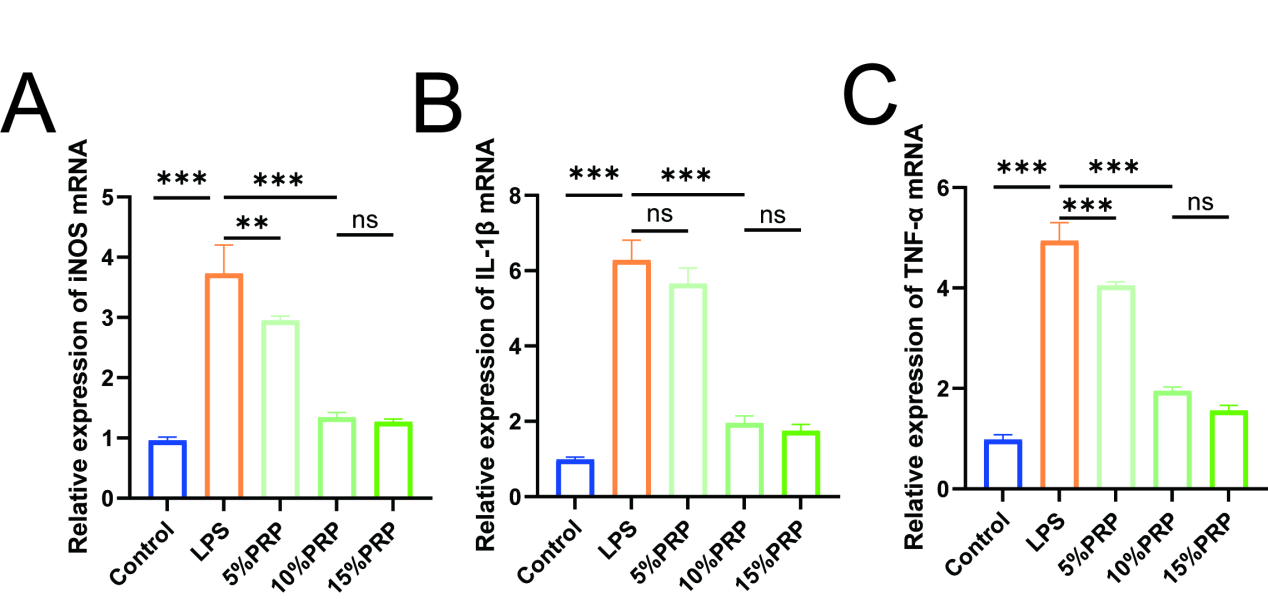
**

**Optimization of the concentration of PRP for LPS-induced M1-type macrophage polarization** (A-C) mRNA expression levels of pro-inflammatory factors (IL-1β and TNF-α) and M1-type macrophage polarization marker(iNOS) in M1-type polarization macrophage detected by RT-qPCR. (ns not significant, ***P* < 0.01, ****P* < 0.001)

**Supplementary figure3:**

**
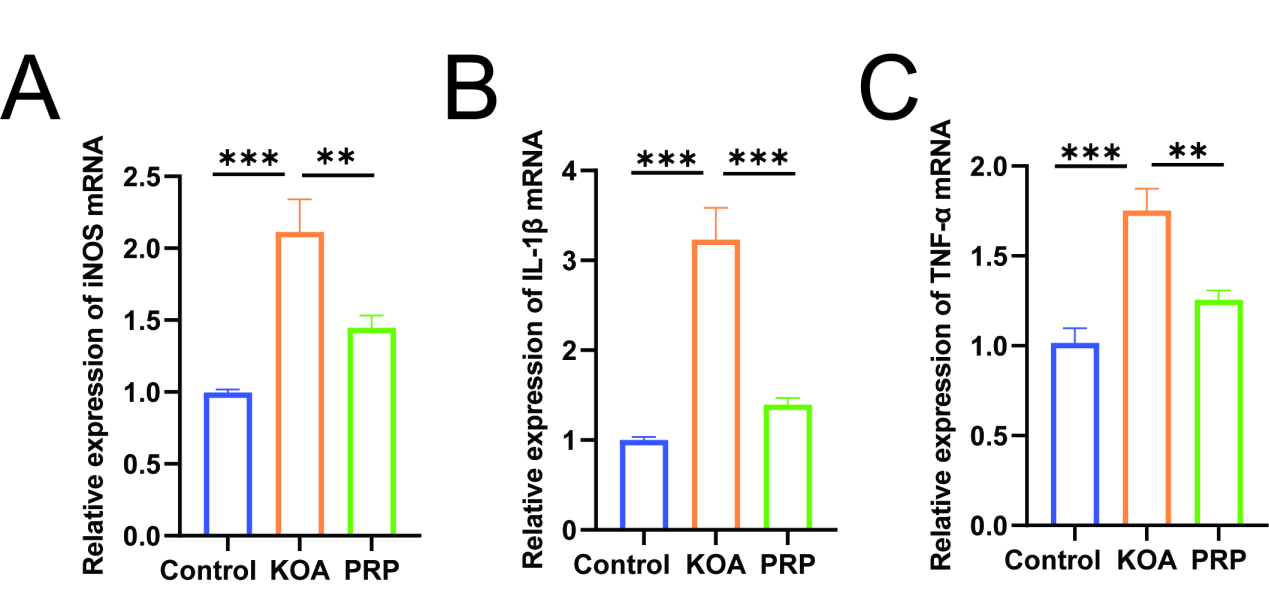
**

**PRP inhibits pro-inflammatory factor secretion in vivo.** (A-C) mRNA expression levels of M1-type macrophage polarization marker(iNOS) and pro-inflammatory factors (IL-1β and TNF-α) in KOA rat model detected by RT-qPCR. (***P* < 0.01, ****P* < 0.001)

**Supplementary figure4:**


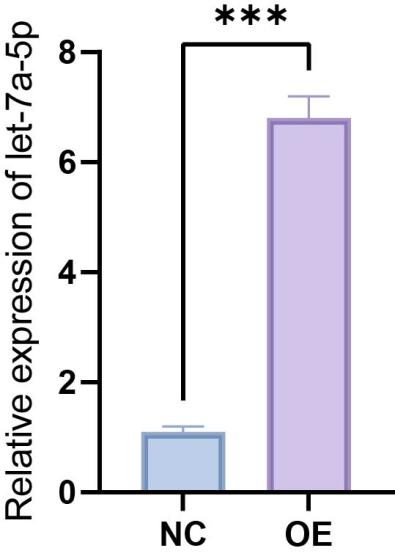


**Verification of the transfection efficiency of let-7a-5p.** The microRNA level of let-7a-5p. (****P* < 0.001)

**Supplementary figure5:**

**
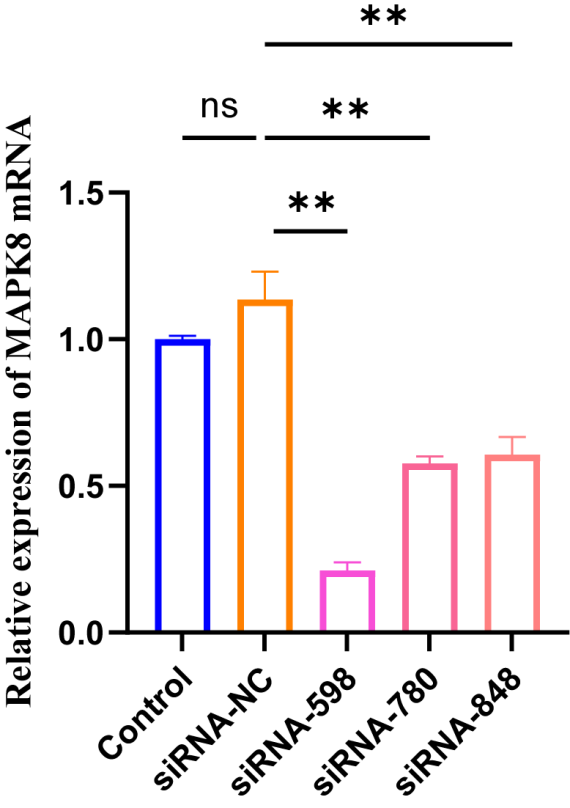
**

**Verification of the transfection efficiency of MAPK8.** The mRNA level of MAPK8. (***P* < 0.01)
